# Post-transplant cyclophosphamide and sirolimus based graft-versus-host disease prophylaxis after allogeneic stem cell transplantation for acute myeloid leukemia

**DOI:** 10.1038/s41409-022-01725-3

**Published:** 2022-06-09

**Authors:** Lorenzo Lazzari, Aitana Balaguer-Roselló, Juan Montoro, Raffaella Greco, Rafael Hernani, Maria Teresa Lupo-Stanghellini, Marta Villalba, Fabio Giglio, Ana Facal, Francesca Lorentino, Manuel Guerreiro, Alessandro Bruno, Ariadna Pérez, Elisabetta Xue, Daniela Clerici, Simona Piemontese, José Luis Piñana, Miguel Ángel Sanz, Carlos Solano, Javier de la Rubia, Fabio Ciceri, Jacopo Peccatori, Jaime Sanz

**Affiliations:** 1 Hematology and Bone Marrow Transplantation Unit, IRCCS Ospedale San Raffaele, Milan, Italy; 2 Department of Hematology, Hospital Universitario y Politécnico La Fe, Valencia, Spain; 3 Department of Hematology, Hospital Clínico Universitario, Valencia, Spain; 4 Department of Medicine, School of Medicine, University of Valencia, Valencia, Spain

**Keywords:** Stem-cell therapies, Acute myeloid leukaemia

## Abstract

Post-transplant cyclophosphamide (PTCy) has emerged as a promising graft-versus-host disease (GvHD) prophylaxis in allogeneic hematopoietic stem cell transplantation (allo-HSCT). However, no studies have reported the efficacy of a GvHD prophylaxis based on PTCy with sirolimus (Sir-PTCy) in patients with acute myeloid leukemia (AML). In this retrospective study, we analyze the use of sirolimus in combination with PTCy, with or without mycophenolate mofetil (MMF), on 242 consecutive adult patients with AML undergoing a myeloablative first allo-HSCT from different donor types, in three European centers between January 2017 and December 2020. Seventy-seven (32%) patients received allo-HSCT from HLA-matched sibling donor, 101 (42%) from HLA-matched and mismatched unrelated donor, and 64 (26%) from haploidentical donor. Except for neutrophil and platelet engraftment, which was slower in the haploidentical cohort, no significant differences were observed in major transplant outcomes according to donor type in univariate and multivariate analysis. GvHD prophylaxis with Sir-PTCy, with or without MMF, is safe and effective in patients with AML undergoing myeloablative allo-HSCT, resulting in low rates of transplant-related mortality, relapse/progression, and acute and chronic GvHD in all donor settings.

## Introduction

Allogeneic hematopoietic stem cell transplantation (allo-HSCT) is an increasingly offered and potentially curative option for patients with acute myeloid leukemia (AML). Regarding GvHD prophylaxis, the success of post-transplant cyclophosphamide (PTCy) in the haploidentical setting has led to the expansion of its use also in recipients of allo-HSCT from HLA-matched sibling donor (MSD) and unrelated donor (MUD) [1, 2]. PTCy combined with additional immunosuppression, mainly calcineurin inhibitors (CNI), has proved to reduce the risk of severe GvHD [3–7]. Looking for a favorable balance between safety and efficacy, encouraging results have recently been reported with CNI-free approaches based on PTCy and sirolimus (Sir-PTCy) [8–12]. Sirolimus demonstrated a favorable toxicity profile, particularly in terms of reduced incidence of acute renal failure [13] and improved regulatory T cells reconstitution [14, 15]. In fact, two large real-life studies have recently reported promising results with Sir-PTCy, with or without mycophenolate mofetil (MMF), as GvHD prophylaxis in patients with hematologic malignancies undergoing allo-HSCT from all donor sources [16, 17]. However, as far as we know, to date, no studies have reported the efficacy of such GvHD prophylaxis with Sir-PTCy restricted to patients with AML.

The aim of this study was to analyze the impact of donor type on transplant outcomes in a large series of patients with AML undergoing myeloablative first allo-HSCT using a uniform platform of GvHD prophylaxis based on Sir-PTCy.

## Patients and methods

### Eligibility criteria and data collection

This is an observational retrospective study. All consecutive patients with AML who received a first allo-HSCT between January 2017 and December 2020 at Hospital Universitari i Politècnic La Fe (Valencia, Spain), Hospital Clínico Universitario (Valencia, Spain), and Ospedale San Raffaele (Milan, Italy) were included in the study.

Data of patients, transplant procedures, toxicity and complications were prospectively collected in all patients and then settled in a computerized database. Clinical charts were additionally reviewed for inconsistent or missing data.

### Donor selection

For donor selection, MSD was the primary option. If not available, a MUD registry search was started. If a matched donor ≥9/10 was not readily available, the best related haploidentical donor (Haplo) was selected according to the following: absence of recipient HLA antibodies against donor antigens, male sex, younger age, matched cytomegalovirus (CMV) serostatus, matched ABO group, and, for female donors, lowest number of prior pregnancies.

### Conditioning regimens and GvHD prophylaxis

Conditioning regimens and GvHD prophylaxis schemes used at the three centers have already been described elsewhere [16, 17]. Sirolimus was started on day +5, with dose modifications to achieve targeted plasma levels between 8 and 14–16 ng/mL, and, in the absence of GvHD or relapse, was gradually tapered from day +90 and finally discontinued on day +150/+180.

All conditioning regimens used in the present study are considered myeloablative according to the latest definitions [18, 19].

Eight patients transplanted at San Raffaele received a single dose of anti-thymocyte globulin 5 mg/Kg on day +5 according to center guidelines due to high graft content of CD3^+^ cells (≥3 ×10^8^/Kg) [20].

### Supportive care

Supportive care measures have already been described elsewhere [16, 17]. CMV prophylaxis with letermovir at a daily dose of 480 mg from day 0 to +100 was implemented at San Raffaele starting from March 2019 in CMV-seropositive recipients [21]. All patients in the Spanish centers received granulocyte colony-stimulating factor (filgrastim) 5 mcg/Kg/day from day +7 until absolute neutrophil count >1 ×10^9^/L for three consecutive days, while this strategy was more recently adopted in San Raffaele (starting from May 2020).

### Definitions

Patients were classified at the time of transplantation according to DRI [22, 23] and the 2017 revised European LeukemiaNet (ELN) genetic risk stratification [24]. Disease status and minimal residual disease (MRD) at the time of transplant were defined according to the ELN recommendations [24, 25]. MRD monitoring was performed with multiparameter flow cytometry or standardized quantitative PCR assays [24, 25]. Comorbidities were evaluated according to the HCT-CI [26]. Post-transplant hematological response and MRD were evaluated at day +30, +90, +180, and +365. Hematologic relapse was defined according to the 2017 ELN recommendations [24]. Myeloid engraftment was defined as the first of 3 consecutive days with neutrophil counts ≥0.5 ×10^9^/L after transplantation. Platelet engraftment was defined as the first of 7 consecutive days with platelet counts ≥20 ×10^9^/L without platelet transfusions. Clinical diagnosis and grading of acute GvHD (aGvHD) and chronic GvHD (cGvHD) were made according to the Glucksberg criteria [27] and the National Institutes of Health consensus criteria [28], respectively. CMV DNAemia and disease [29], Epstein-Barr virus (EBV) DNAemia [30], invasive fungal infection [31], and BK polyomavirus-associated hemorrhagic cystitis [32] were diagnosed according to consensus criteria. Toxicities were graded using NCI Common Toxicity Criteria version 5.0 except for SOS, which was graded using the EBMT criteria [33].

### Statistical analysis

Endpoints of the study were leukemia-free survival (LFS), overall survival (OS), graft-versus-host-free/relapse-free survival (GRFS), transplant-related mortality (TRM), cumulative incidence of relapse/progression (CIR), cumulative incidence of neutrophils and platelet engraftment, and cumulative incidence of aGvHD and cGvHD. Last follow-up was considered the 1st of July 2021. Unadjusted time-to-event analyses were performed using the Kaplan-Meier estimate [34], and, for comparisons, the log-rank tests [35]. LFS was defined as the time to death or relapse/progression, whichever came first. OS was defined as the time from transplant to death from all causes. For the analysis of GRFS, grade III-IV aGvHD, moderate-to-severe cGvHD, relapse, graft failure, and death were considered uncensored events. The probabilities of engraftment, TRM, GvHD, CIR, SOS, CMV DNAemia, and hemorrhagic cystitis were calculated using the competing risk structure [34, 35]. Competing risks data were considered as follows: for myeloid and platelet engraftment, early death or second allo-HSCT with no evidence of engraftment; for aGvHD, relapse before aGvHD or death without aGvHD before day +100; for cGvHD, death without development of cGvHD or relapse before the development of cGvHD; for TRM, relapse; for CIR, death with no previous relapse; and for other post-transplant events, death or relapse with no previous event. Patient and transplantation characteristics according to donor type were compared using the chi-square test for categorical variables and the Wilcoxon rank-sum test for continuous variables. A 95% confidence interval (95% CI) was considered. A p-value lower than 0.05 was interpreted as significant. The Cox proportional hazard model or the Gray method for competing events were used for multivariate analysis [36]. All factors known to influence outcome and factors associated with a univariate analysis *p*-value <0.10 were first included in the model; a stepwise backward procedure was used with a cut-off significance level of 0.10 for deleting factors from the model. The type I error rate was fixed at 0.05 for determination of factors associated with time to event. Statistical analyses were performed using R statistical software version 4.0.4 (R Development Core Team, Vienna, Austria) and SPSS version 25.0 (IBM Corporation, Armonk, NY).

## Results

Patient, disease and transplant characteristics of the overall population and according to donor type are summarized in Table 1 and Table 2. Briefly, a total of 242 AML patients were included in the study, of which 77 (32%) received an allo-HSCT from MSD, 101 (42%) from MUD—including 16 9/10-HLA-mismatched unrelated donor (MMUD)—and 64 (26%) from Haplo. Median follow-up of surviving patients was 25 months (range, 6–52), with no differences according to donor type. MSD, MUD, and Haplo recipients did not differ with respect to patient and disease characteristics, except for a higher age in the MSD cohort (*p* = 0.02) and a slightly greater proportion of adverse genetic category in MUD recipients (*p* = 0.045). Regarding transplant characteristics, donor age was also higher in the MSD cohort (*p* < 0.0001).

**Table 1:** Patient and disease characteristics according to donor type.

Characteristics	Total	MSD	MUD	Haplo	*P*
No. of patients, no. (%)	242	77 (32)	101 (42)^a^	64 (26)	
Recipient age in years, median (range)	55 (16–74)	51 (16–69)	57 (18–73)	56 (17–74)	0.02
Sex, no. (%)					0.4
Male	147 (61)	50 (65)	57 (56)	40 (64)	
Female	95 (39)	27 (35)	44 (44)	24 (36)	
Type of AML, no. (%)					0.4
de novo	214 (88)	71 (92)	87 (86)	56 (87)	
tAML/sAML	28 (12)	6 (8)	14 (14)	8 (13)	
Disease status at allo-HSCT, no. (%)					0.4
CR1	161 (66)	55 (73)	69 (68)	37 (57)	
≥CR2	45 (19)	11 (14)	18 (18)	16 (25)	
Active disease	36 (15)	11 (13)	14 (14)	11 (18)	
ELN 2017 classification^b^, no. (%)					0.045
Favorable	43 (18)	20 (26)	10 (11)	13 (19)	
Intermediate	102 (43)	33 (43)	40 (41)	29 (46)	
Adverse	95 (39)	24 (31)	49 (49)	22 (35)	
Disease risk index, no. (%)					0.8
Low	26 (11)	11 (14)	7 (8)	8 (12)	
Intermediate	123 (51)	40 (52)	50 (49)	33 (52)	
High	78 (32)	22 (29)	37 (36)	19 (30)	
Very high	15 (6)	4 (5)	7 (7)	4 (6)	
MRD status for patients in CR, no. (%)					0.15
Positive	63 (26)	23 (30)	22 (22)	18 (28)	
Negative	77 (32)	30 (39)	29 (29)	18 (28)	
Missing	66 (27)	13 (17)	36 (36)	17 (27)	
Not applicable	36 (15)	10 (14)	15 (13)	11 (17)	
Prior auto-HSCT, no. (%)	10 (4)	4 (5)	3 (3)	3 (5)	0.9
HCT-CI, no. (%)					0.6
0	71 (29)	20 (26)	30 (29)	21 (33)	
1–2	67 (28)	26 (34)	27 (27)	14 (22)	
≥3	104 (43)	31 (40)	44 (44)	29 (45)	
Median follow-up, months (range)	25 (6–52)	24 (7–50)	19 (6–52)	23 (7–51)	0.4

*MSD* matched sibling donor, *MUD* matched unrelated donor, *Haplo* haploidentical donor, *AML* acute myeloid leukemia, *tAML/sAML* transformed AML/secondary AML, *allo-HSCT* allogeneic hematopoietic stem cell transplant, *CR* complete remission, *ELN* European LeukemiaNet, *MRD* minimal residual disease, *auto-HSCT* autologous hematopoietic stem cell transplant, *HCT-CI* hematopoietic cell transplant-comorbidity index.

^a^16 mismatched unrelated donors (MMUD) are included.

^b^1 missing.

**Table 2:** Transplant characteristics according to donor type.

Characteristics	Total	MSD	MUD	Haplo	*P*
No. of patients, no. (%)	242	77 (32)	101 (42)^a^	64 (26)	
Donor age in years, median (range)	37 (12–68)	48 (12–68)	30 (18–59)	37 (16–67)	<0.0001
ABO blood group mismatch, no. (%)					0.2
Major	48 (20)	14 (18)	27 (27)	7 (11)	
Minor	55 (23)	17 (22)	22 (22)	16 (26)	
Female donor to male recipient, no. (%)	52 (22)	23 (30)	15 (15)	14 (22)	0.2
Donor-recipient CMV serostatus, no. (%)					0.002
Positive/Positive	156 (65)	57 (75)	51 (50)	48 (76)	
Positive/Negative	13 (5)	4 (5)	8 (8)	1 (2)	
Negative/Positive	60 (25)	11 (15)	38 (37)	11 (18)	
Negative/Negative	12 (5)	4 (5)	5 (5)	3 (5)	
Conditioning regimen, no. (%)					0.002
Busulfan-based:	116 (48)	47 (61)	49 (48)	20 (32)	
TB3F	53 (22)	2 (3)	29 (28)	8 (13)	
TB2F	39 (16)	21 (27)	20 (20)	12 (19)	
Bu-Flu	24 (10)	24 (31)	0	0	
Treosulfan-based:	126 (52)	30 (39)	52 (52)	44 (68)	
Treo-Flu	38 (16)	7 (9)	16 (16)	15 (24)	
Thio-Treo-Flu	27 (11)	1 (1)	1 (2)	25 (38)	
Treo-Flu-Mel	58 (24)	20 (26)	35 (34)	3 (5)	
Treo-Flu-TBI	3 (1)	2 (3)	0	1 (1)	
GvHD prophylaxis, no. (%)					<0.0001
PTCy + Sir + MMF	206 (85)	48 (62)	98 (97)	60 (94)	
PTCy + Sir	28 (12)	28 (36)	0	0	
PTCy + Sir + /- MMF + ATG	8 (3)	1 (1)	3 (3)	4 (6)	
Stem cell source, no. (%)					0.08
Bone marrow	6 (3)	1 (1)	1 (1)	4 (6)	
Peripheral blood	236 (97)	76 (99)	101 (99)	59 (94)	
Cell dose infused, median (range)					
TNC/Kg x10^8^	7.8 (1.6–35.8)^b^	7.8 (1.6–35.8)	7.8 (2–17)	8.6 (2.2–28.4)	0.8
CD34^+^/Kg x10^6^	6.8 (0.7–23)	6.6 (0.7–18.8)	6.7 (1.2–23)	7 (2.2–13.2)	0.6
CD3^+^/Kg x10^8^	2.3 (0.2–7.8)^c^	2.5 (0.4–7)	2.2 (0.2–7.8)	2.4 (0.3–7.6)	0.1
Median follow-up, months (range)	25 (6–52)	24 (7–50)	19 (6–52)	23 (7–51)	0.3

*MSD* matched sibling donor, *MUD* matched unrelated donor, *Haplo* haploidentical donor, *CMV* cytomegalovirus, *TBF* thiotepa, busulfan and fludarabine, *Bu-Flu* busulfan and fludarabine, *Treo-Flu* treosulfan and fludarabine, *Thio-Treo-Flu* thiotepa, treosulfan and fludarabine, *Treo-Flu-Mel* treosulfan, fludarabine and melphalan, *Treo-Flu-TBI* treosulfan, fludarabine and TBI 4 Gy, *GvHD* graft-versus-host disease, *PTCy* post-transplant cyclophosphamide, *Sir* sirolimus, *MMF* mycophenolate mofetil, *TNC* total nucleated cell.

^a^16 mismatched unrelated donors (MMUD) are included.

^b^43 missing.

^c^31 missing.

### Engraftment

Eight patients died without evidence of myeloid engraftment, 5 in the MUD cohort (4 infections and 1 acute renal failure), 2 in the Haplo cohort (cerebral hemorrhage and disease progression), and one from MSD (infection). Primary graft failure was only observed in 2 haploidentical recipients with high titer of anti-HLA donor specific antibodies; both patients were successfully retransplanted from the same donor after a desensitization therapy. The remaining 232 patients achieve neutrophil engraftment at a median time of 19 days (range, 13–51). Median time to neutrophil recovery for MSD, MUD, and Haplo was 17 days (range, 13–51), 19 days (range, 13–43), and 21 days (range, 13–49), respectively. No secondary graft failure was observed. All engrafted patients achieved full donor chimerism. Ten patients (2 MSD, 1 MUD, and 7 Haplo) underwent a CD34^+^ selected boost for secondary poor graft function. Cumulative incidence of neutrophil recovery at 60 days was lower in haploidentical recipients (*p* = 0.003) (Table 3) and higher (97%) and faster (16 days) in those patients receiving a higher CD34^+^ graft cell content (beyond percentile 75, 7.8 ×10^6^/Kg) (*p* = 0.008).

**Table 3:** Univariate analysis of transplants outcomes according to donor type.

Outcomes^a^	Overall	MSD	MUD^b^	Haplo	*P*
ANC engraftment, CI at 60 days (%)	96 (93–98)	97 (94–100)	94 (90–99)	92 (85–99)	0.003
Platelet engraftment, CI at 180 days (%)	95 (93–98)	98 (95–100)	95 (90–100)	93 (86–100)	0.01
aGvHD, % (95% CI)					
grade II-IV	21 (16–26)	18 (12–28)	21 (14–29)	23 (14–34)	0.6
grade III-IV	7 (4–11)	6 (2–14)	7 (3–13)	8 (3–16)	0.9
cGvHD, % (95% CI)					
any grade	45 (38–52)	50 (37–62)	37 (27–47)	50 (36–64)	0.1
moderate-to-severe	28 (22–34)	35 (24–46)	25 (17–35)	24 (14–36)	0.3
severe	8 (5–12)	7 (3–14)	8 (4–15)	10 (4–20)	0.8
TRM, % (95% CI)	13 (9–18)	8 (3–15)	14 (8–22)	19 (10–30)	0.1
CIR, % (95% CI)	16 (11–21)	20 (11–31)	16 (9–24)	9 (4–18)	0.4
LFS, % (95% CI)	71 (65–77)	72 (59–82)	71 (61–80)	71 (57–81)	0.6
OS, % (95% CI)	74 (68–80)	78 (68–84)	73 (63–82)	72 (58–82)	0.3
GRFS, % (95% CI)	47 (40–53)	46 (34–57)	48 (38–58)	46 (32–58)	0.9

*aGvHD* acute graft-versus-host disease, *cGvHD* chronic graft-versus-host disease, *ANC* neutrophil, *95% CI* 95% confidence interval, *TRM* transplant-related mortality, *CIR* cumulative incidence of relapse/progression, *LFS* leukemia-free survival, *OS* overall survival, *GRFS* graft-versus-host-free/relapse-free survival.

^a^ANC and platelet engraftment: cumulative incidence at 60 and 180 days, respectively; acute GvHD: 100-day cumulative incidence; chronic GvHD, TRM, CIR, LFS, OS, and GRFS: survival probability at 2 years.

^b^16 mismatched unrelated donors (MMUD) are included: outcomes for this group of patients were not statistically different from that of recipients of MUD allo-HSCT (data not shown).

Median time of platelet recovery was 25 days (range, 11–313) for the overall population and 20 days (range, 17–259) for MSD, 26 days (range, 11–257) for MUD, and 33 days (range, 17–313) for Haplo. Cumulative incidence of platelet recovery was lower in the Haplo cohort (*p* = 0.01) (Table 3).

There was no statistical difference in immune reconstitution data according to the type of donor (Table S1).

### GvHD

There were 9 aGvHD that were diagnosed after day 100 without any difference according to donor source (2 MSD, 4 MUD, 3 Haplo; *p* = 0.7), with a median time to diagnosis of 129 days (range, 105–204); 3 patients developed late onset aGvHD after infusion of a CD34-selected boost administered due to poor graft function.

Fifteen patients developed an overlap cGvHD in our series at a median time of 155 days (range, 79–538). No statistical difference was observed according to donor groups (4 MSD, 3, 8 Haplo; *p* = 0.2). Three of these patients developed the condition after infusion of a CD34-selected stem cell boost for poor graft function, and two patients after respectively 3 and 4 months of therapy with a FLT3-inhibitor.

**Fig. 1:**
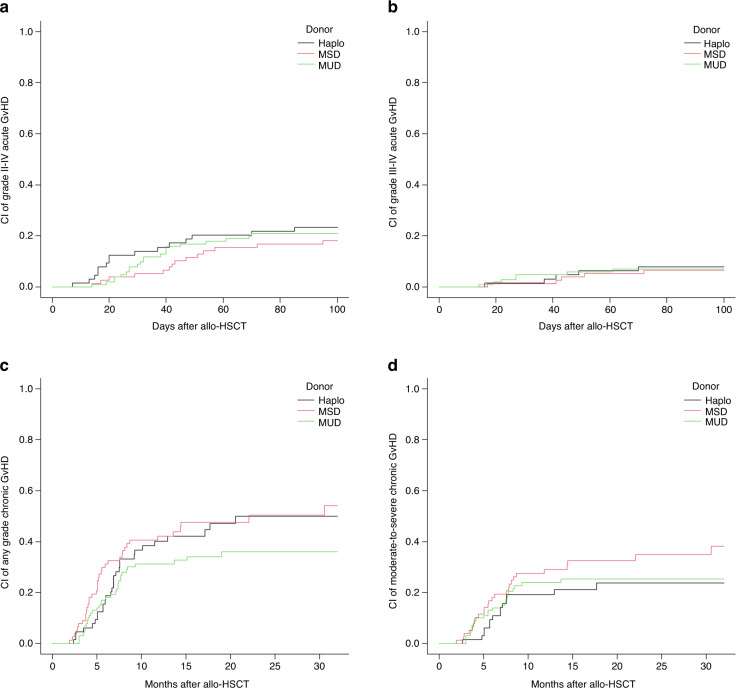
Incidence of acute and chronic graft-versus-host disease (GvHD). Cumulative incidence (CI) of acute GvHD grade II-IV (**a**) and grade III-IV (**b**), and CI of chronic GvHD any grade (**c**) and moderate-to-severe (**d**) according to donor type. Haplo haploidentical donor, MSD matched sibling donor, MUD matched unrelated donor.

Cumulative incidence of aGvHD and cGvHD according to donor type are displayed in Table 3. There was no statistical difference in the incidence and pattern of acute or chronic GvHD according to donor type (Fig. 1).

Overall, aGvHD occurred in 100 (41%) patients at a median time of 37 days (range, 8–170): 44 (45%) grade I, 36 (35%) grade II, 15 (15%) grade III, and 5 (5%) grade IV. No variables were associated with an increased risk aGvHD in multivariate analysis.

Overall, cGvHD occurred in 100 (41%) patients at a median time of 173 days (range, 59–930). According to the NIH classification, 35 (35%) were mild, 46 (46%) moderate, and 19 (19%) severe. Results of multivariate analysis are displayed in Table 4.

**Table 4:** Multivariate analysis of significant transplants outcomes.

Outcomes	Variables	95% CI per HR		HR	*P*
		Lower	Upper		
Any grade cGvHD	Donor age ≥40 years	1.15	3.79	2.1	0.01
	CD34^+^ graft content (>7.8 ×10^6^/Kg)	1.07	2.37	1.54	0.04
Moderate-to-severe cGvHD	Donor age ≥40 years	1.28	6.96	2.99	0.01
	CD34^+^ graft content (>7.8 ×10^6^/Kg)	1.06	2.94	1.76	0.03
	Female-to-male sex mismatch	1.06	3.06	1.81	0.03
TRM	Patient age ≥55 years	1.24	6.26	2.79	0.01
	Active disease at allo-HSCT	1.09	7.85	2.93	0.03
CIR	Very high DRI	1.92	4.68	2.85	0.03
	Active disease at allo-HSCT	2.13	16.25	5.89	0.001
	MRD positivity at allo-HSCT	1.15	7.83	3.0	0.02
OS	Very high DRI	1.3	97.6	11.2	0.03
	Active disease at allo-HSCT	1.53	9.16	3.75	0.004
	HCT-CI ≥3	1.05	2.88	1.74	0.03
LFS	Active disease at allo-HSCT	2.58	10.52	5.2	0.03
	CR status without MRD data	1.03	4.13	2.06	<0.001
	HCT-CI ≥3	1.03	2.63	1.65	0.034
GRFS	Donor age ≥40 years	1.06	2.81	1.73	0.03
	High DRI	1.23	5.55	2.61	0.01
	Very high DRI	2.48	15.05	6.11	<0.001

*CI* confidence interval, *HR* hazard ratio, *aGvHD* acute graft-versus-host disease, *cGvHD* chronic graft-versus-host disease, *TRM* transplant-related mortality, *CIR* cumulative incidence of relapse/progression, *OS* overall survival, *LFS* leukemia-free survival, *GRFS* graft-versus-host-free/relapse-free survival, *allo-HSCT* allogeneic hematopoietic stem cell transplantation, *HCT-CI* hematopoietic cell transplantation-specific comorbidity index, *CR* complete remission, *MRD* minimal residual disease, *DRI* disease risk index.

### Post-transplant complications

The main post-transplant complications are detailed in Table S2. There were no differences in the cumulative incidence of these complications according to the type of donor. In multivariate analysis, busulfan-based conditioning regimen was identified as risk factor for mucositis (p < 0.0001) and hemorrhagic cystitis (p < 0.0001). Patients receiving treosulfan-based conditioning regimen had a significantly lower incidence of SOS (0.8% versus 5%; p = 0.02) in univariate analysis, but this result was not confirmed in multivariate analysis.

### TRM, CIR, and survival

**Fig. 2:**
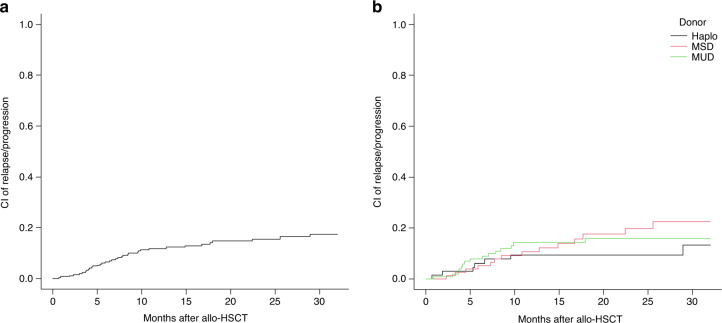
Incidence of relapse/progression. Cumulative incidence (CI) of relapse/progression of the overall cohort (**a**) and according to donor type (**b**). Haplo haploidentical donor, MSD matched sibling donor, MUD matched unrelated donor.

Thirty-four patients died without prior relapse or progression at a median time of 82 days (range, 5–1187). All causes of death according to donor type are shown in Table S3. According to the type of donor, differences in TRM were not statistically significant (Table 3 and Fig. 2). However, MSD recipients had a lower TRM compared with alternative donors in univariate analysis (8% versus 16%, *p* = 0.046), but this was not confirmed in multivariate analysis.

Thirty-eight patients relapsed or progressed after allo-HSCT at a median time of 7 months (range, 1–43). Extramedullary relapse occurred in 15 patients, either isolated (*n* = 7) or in combination with marrow relapse (*n* = 8), with central nervous system being the most common site involved (*n* = 5). Patients with extramedullary relapse did not have different characteristics than those with hematological relapse. One additional patient was diagnosed with HLA loss relapse 6 months after Haplo. We did not observe significant differences in the pattern of relapses and the CIR according to the type of donor (Table 3 and Fig. 2).

There were no statistically significant differences in OS, LFS and GRFS according to the type of donor (Table 3). Results of univariate analysis of survival outcomes are showed in Table 5.

**Table 5:** Univariate analysis of significant survival outcomes.

Variables	2-year rate (95% CI)	*P*
Leukemia-free survival		
DRI:		
Low	87 (66–96)	<0.001
Intermediate	75 (65–82)	
High	67 (53–77)	
Very high	29 (8–55)	
2017 ELN risk:		
Favorable	76 (59–86)	0.01
Intermediate	76 (66–84)	
Adverse	63 (52–72)	
Disease status at allo-HSCT:		
Active disease	40 (21–58)	<0.001
CR/pos MRD	73 (59–83)	
CR/neg MRD	82 (71–90)	
CR/no MRD	71 (58–81)	
HCT-CI:		
<3	77 (70–85)	0.02
≥3	62 (53–74)	
MRD status at 90 days:		
Positive	65 (46–91)	0.04
Negative	86 (79–94)	
Overall survival		
DRI:		
Low	96 (75–99)	<0.001
Intermediate	77 (68–84)	
High	70 (57–80)	
Very high	29 (8–54)	
2017 ELN risk:		
Favorable	79 (62–89)	0.03
Intermediate	79 (69–86)	
Adverse	67 (55–76)	
Disease status at allo-HSCT:		
Active disease	43 (25–60)	<0.001
CR/pos MRD	78 (64–87)	
CR/neg MRD	87 (77–93)	
CR/no MRD	74 (61–83)	
HCT-CI:		
<3	79 (71–86)	0.03
≥3	69 (60–79)	
Graft-versus-host-free/relapse-free survival		
DRI:		
Low	58 (37–74)	0.002
Intermediate	53 (44–62)	
High	40 (28–52)	
Very high	8 (5–30)	
2017 ELN risk:		
Favorable	55 (38–69)	0.02
Intermediate	52 (42–62)	
Adverse	37 (27–47)	
Disease status at allo-HSCT:		
Active disease	28 (14–44)	0.01
CR/pos MRD	43 (30–56)	
CR/neg MRD	55 (42–66)	
CR/no MRD	51 (14–44)	

*y* years, *95% CI* 95% confidence interval, *HR* hazard ratio, *DRI* disease risk index, *ELN* European LeukemiaNet, *allo-HSCT* allogeneic hematopoietic stem cell transplant, *HCT-CI* hematopoietic cell transplantation-specific comorbidity index, *CR* complete remission, *MRD* minimal residual disease.

Results of multivariate analysis are displayed in Table 4.

## Discussion

The present study shows that Sir-PTCy, with or without MMF, for GvHD prophylaxis is safe and effective in patients with AML undergoing myeloablative allo-HSCT not only from Haplo but also from MSD and MUD, resulting in low rates of TRM, CIR, aGvHD, and cGvHD in all transplant settings. Patient age (≥55 years) and active disease status at allo-HSCT were the only independent risk factors found for TRM, with the latter along with MRD positive status being also associated with a higher risk of relapse/progression. The lack of statistically significant differences in TRM and relapse/progression according to the type of donor translated into an absence of significant differences also in OS, LFS, and GRFS.

Although non-randomized, this study included a real-life unselected population of AML patients who underwent first allo-HSCT with a similar CNI-free GvHD prophylaxis in three European centers during the same study period. Recently reported studies have demonstrated that this approach is not only feasible and effective after Haplo, but also in allo-HSCT from MSD and MUD for patients with a variety of hematologic malignancies [8–12, 16, 17]. However, to the best of our knowledge, this is the first to compare the results of such GvHD prophylaxis across different donor types in AML. Since donor selection was based primarily on availability and not on patient or disease characteristics, there were no significant differences between the three cohorts in most baseline features, with the exception of younger age in the MSD cohort and higher proportion of adverse cytogenetics in those transplanted from MUD. Regarding transplant characteristics, some additional differences were also observed. While GvHD prophylaxis was relatively homogeneous (Sir-PTCy-MMF) in the vast majority of patients (88%), 28 patients transplanted from MSD received Sir-PTCy without MMF. It should also be noted that the proportion of treosulfan-based conditioning was higher in the haploidentical cohort, while the proportion of busulfan-based conditioning and donor age were higher in the MSD cohort.

The relatively low rates of grade II-IV and grade III-IV aGvHD, as well as moderate-to-severe cGvHD, are similar to those previously reported separately in hematologic malignancies with this GvHD prophylaxis by the Italian [17] and Spanish [16] centers participating in the present study. Our results were apparently better than that reported with standard CNI-based GvHD prophylaxis [37–42] and comparable to those reported with PTCy in combination with CNI [1, 3, 43–46], particularly considering that most transplants in our series were performed with peripheral blood as stem cell source (MSD and MUD, 99%; Haplo, 94%). Interestingly, the type of donor did not have a significant impact on the incidence of aGvHD and cGvHD, with donor age (older than 40 years) and a higher CD34^+^ content in the graft (beyond percentile 75, 7.8 × 10^6^/Kg) being the main variables associated with an increased risk of cGvHD in multivariate analysis.

In addition to the acceptable incidence of aGvHD and cGvHD, encouraging results were obtained in terms of CIR, TRM, OS, LFS, and GRFS when compared with other series that used PTCy in patients with AML [3, 4, 6, 38, 43], without significant differences between donor types. Notably, in our study a slower recovery of neutrophil and platelet was observed in the Haplo cohort, which, in contrast with reports from other authors [47, 48], did not translate into a higher incidence of TRM or post-transplant complications. In the same group, we documented two cases of primary graft failure and seven of secondary poor graft function requiring a CD34-selected boost, two conditions already described to be more associated with mismatched allo-HSCT [49, 50].

The fact that the median time to neutrophil and platelet recovery appears somewhat longer in our study than with CNI-based GvHD prophylaxis is likely due to PTCy. In fact, a recently reported randomized phase III trial that compared two CNI-free approaches (CD34-selected grafts and PTCy) to standard CNI-based GvHD prevention, with tacrolimus and methotrexate, showed that grafting after PTCy was slower compared to the others [51].

Our results are partially in contrast with a recent registry-based study in which patients with AML undergoing Haplo allo-HSCT displayed a higher rate of aGvHD and TRM, coupled with a lower incidence of relapse, compared to recipients of MSD and MUD [6]. In fact, other comparative studies have also suggested a superior graft-versus-leukemia effect in Haplo compared to MSD transplants for high-risk AML [52–54], but we were unable to demonstrate this effect in the present study. Another recent analysis that compared recipients of MUD to those of Haplo reported better outcomes with MUD in transplants with reduced-intensity conditioning but not with myeloablative regimens [47].

The relatively low incidence of relapse (16% at 2 years) reported in the present study, despite the considerable number of patients transplanted with active disease (15%) or ≥CR2 (19%), should be highlighted. Although conflicting results have been reported by other authors regarding the impact of conditioning intensity on the risk of relapse [55–58], our GvHD prophylaxis in combination with myeloablative conditioning showed potent antileukemic efficacy and was well tolerated even in older patients with comorbidities. Other alternative or complementary explanations to consider could be the possible synergistic effect of Sir-PTCy on the in vivo expansion of regulatory T cells in order maintain a balance between GvHD and disease recurrence [15, 59] or the potential antileukemic effect of sirolimus [60]. Another indirect proof of the graft-versus-leukemia effect displayed with our approach could be the relatively high proportion of extramedullary relapses (15 out of 38 relapses), as an expression of the mechanisms adopted by leukemic cells to evade immune recognition. We were unable to find a correlation of extramedullary relapses with any genomic characteristic or MRD status (data available in only 140 patients).

In addition to the well-known prognostic factors such as DRI, 2017 ELN risk classification, and disease status at allo-HSCT, we also confirmed in our study the independent prognostic impact of pretransplant MRD status on the risk of relapse [61, 62]. Although very high DRI and active disease status at transplant were unfavorable variables for relapse risk and survival, a considerable fraction of these very high-risk patients could be saved with our strategy showing a 2-year LFS of respectively 29% and 40% (Table 5).

Furthermore, we were unable to detect any impact on engraftment, GvHD, and infections when MMF was omitted from GvHD prophylaxis, but it should be noted that this group consisted of only 28 patients, which represents 11% of the total and a third of those transplanted from MSD, a very small sample size to analyze its impact.

In conclusion, myeloablative allo-HSCT for AML with Sir-PTCy, with or without MMF, as GvHD prophylaxis resulted in a low incidence of relapse/progression and TRM, acceptable rates of aGvHD and cGvHD, and good survival outcomes for all donor types. Despite the absence of major differences in transplant outcomes by donor type using this GvHD prophylaxis, a possible change in donor selection algorithm for AML patients in need of an allo-HSCT should still await confirmation of our results in prospective controlled studies. Future studies should clarify whether other donor characteristics should take priority over donor type under certain circumstances in the setting of Sir-PTCy GvHD prophylaxis.

## Supplementary information

Supplementary material

## Data Availability

The dataset generated and analyzed during the current study is available in the Figshare repository, 10.6084/m9.figshare.19688673.
