# Supplementary material for: Post-transplant cyclophosphamide and sirolimus based graft-versus-host disease prophylaxis after allogeneic stem cell transplantation for acute myeloid leukemia
Source: Bone Marrow Transplant. 2022 Jun 9;57(9):1389–98. doi: 10.1038/s41409-022-01725-3 (PMC9439951; doi:10.1038/s41409-022-01725-3)
Supplement: Supplementary file 1 — Supplementary material [file 41409_2022_1725_MOESM1_ESM.docx]

Supplementary material for

**Post-transplant cyclophosphamide and sirolimus based graft-versus-host disease prophylaxis after allogeneic stem cell transplantation for acute myeloid leukemia**

**Supplementary Tables**

**Table S1.** Immune reconstitution data after allo-HSCT according to donor type.

|  | **CD3^#^** | **CD4^#^** | **CD8^#^** | **CD19^#^** | **CD56^#^** |
| --- | --- | --- | --- | --- | --- |
| **+3 months*** | | | | | |
| **MSD** | 625 | 213 | 396 | 6 | 153 |
| **MUD** | 730 | 218 | 438 | 14 | 150 |
| **Haplo** | 684 | 205 | 480 | 17 | 129 |
| **+6 months*** | | | | | |
| **MSD** | 878 | 239 | 577 | 80 | 153 |
| **MUD** | 1248 | 303 | 743 | 96 | 174 |
| **Haplo** | 946 | 295 | 653 | 33 | 185 |
| **+12 months*** | | | | | |
| **MSD** | 1186 | 381 | 853 | 161 | 209 |
| **MUD** | 1382 | 373 | 933 | 274 | 227 |
| **Haplo** | 1509 | 460 | 1023 | 200 | 224 |

*Missing: 102 patients at +3 months, 98 at +6 months, and 90 at +12 months.

^#^ cell/uL (median)

**Abbreviations:** MSD, matched sibling donor; MUD, matched unrelated donor; Haplo, haploidentical donor.

**Table S2.** Post-transplant complications.

|  | **Overall,**  no. (%) | **Days,**  median (range) | **Cumulative incidence at 100 days,**  % (95% CI) | **Risk factors (Multivariate analysis)** | | |
| --- | --- | --- | --- | --- | --- | --- |
|  |  |  |  | Variables | HR (95% CI) | *P* |
| **Oral mucositis:**  Grade I  Grade II  Grade III  Grade IV | 134 (55.5)  31 (23)  50 (37)  48 (36)  5 (4) | 8 (7-27) | 33 (27-39) | Busulfan-based conditioning | 4.8  (2.8-8) | <0.0001 |
| **Hemorrhagic cystitis:**  Grade I  Grade II  Grade III  Grade IV | 67 (27)  28 (42)  21 (31)  16 (24)  2 (3) | 18 (12-28) | 27 (21-32) | Busulfan-based conditioning | 2.91  (1.72-4.93) | <0.0001 |
| **SOS:**  Mild  Moderate  Severe  Very Severe | 8 (3)  3 (37.5)  0  2 (25)  3 (37.5) | 41 (21-235) | 3 (0.8-5) | NA | | |
| **TMA** | 6 (2) | 122 (44-311) | 1 (0.2-3) | NA | | |
| **CMV reactivation*** | 76 (31) | 47 (1-558) | 31 (26-37) | No CMV prophylaxis  No MSD  D–/R+ | 2.79  (1.5-5.2)  2.27  (1.2-4.3)  2.94  (1.6-6.1) | 0.001  0.01  <0.001 |
| **CMV disease*** | 9 (4) | 58 (29-269) | 3.8 (2-7) | NA | | |
| **EBV reactivation*** | 8 (3) | 138 (26-189) | 4.1 (2-7) | NA | | |
| **IFI*** | 23 (9) | 80 (5-1233) | 4.1 (2-7) | NA | | |

*Cumulative incidence at 2 years.

**Abbreviations:** CI, confidence interval; HR, Hazard Ratio; SOS, sinusoidal Obstruction Syndrome; TMA, thrombotic microangiopathy; CMV, Cytomegalovirus; D, donor; R, recipient; EBV, Epstein Barr virus; IFI, invasive fungal infection; NA, not applicable.

**Table S3.** Causes of death according to donor type.

|  | **Overall**  N = 242 | **MSD**  N = 77 | **MUD***  N = 101 | **Haplo**  N = 64 |
| --- | --- | --- | --- | --- |
| **Relapse or progression,** no. (%) | 28 (12) | 10 (13.9) | 11 (11.5) | 7 (11.5) |
| **Non-relapse causes,** no. (%) | 34 (14) | 6 (8.3) | 16 (16.6) | 12 (19) |
| Infections  Bacterial  Fungal  Viral  Others or mixed  Unknown | 20 (58.8)  5  3  7  2  3 | 4 (66.7)  1  1  2  0  0 | 8 (50)  3  2  2  0  1 | 8 (66.7)  1  0  3  1  3 |
| GvHD | 6 (17.8) | 2 (33.3) | 3 (18.9) | 1 (8.3) |
| Interstitial pneumonitis | 2 (5.9) | 0 | 2 (12.5) | 0 |
| SOS | 1 (2.9) | 0 | 1 (6.2) | 0 |
| Hemorrhage | 1 (2.9) | 0 | 0 | 1 (8.3) |
| Acute renal failure | 1 (2.9) | 0 | 1 (6.2) | 0 |
| Unknown cause | 3 (8.8) | 0 | 1 (6.2) | 2 (16.7) |

*16 mismatched unrelated donors (MMUD) are included.

**Abbreviations:** MSD, matched sibling donor; MUD, matched unrelated donor; Haplo, haploidentical donor; GvHD, graft-versus-host disease; SOS, sinusoidal obstruction syndrome.
